# Comprehensive Analysis of Secondary Dental Root Canal Infections: A Combination of Culture and Culture-Independent Approaches Reveals New Insights

**DOI:** 10.1371/journal.pone.0049576

**Published:** 2012-11-12

**Authors:** Annette Carola Anderson, Elmar Hellwig, Robin Vespermann, Annette Wittmer, Michael Schmid, Lamprini Karygianni, Ali Al-Ahmad

**Affiliations:** 1 Department of Operative Dentistry and Periodontology, Albert-Ludwigs-University, Freiburg, Germany; 2 Department of Hygiene and Microbiology, Albert-Ludwigs-University, Freiburg, Germany; 3 Michael Schmid, Research Unit Microbe-Plant Interactions, Helmholtz Zentrum München, German Research Center for Environmental Health, Neuherberg, Germany; University of Toronto, Canada

## Abstract

Persistence of microorganisms or reinfections are the main reasons for failure of root canal therapy. Very few studies to date have included culture-independent methods to assess the microbiota, including non-cultivable microorganisms. The aim of this study was to combine culture methods with culture-independent cloning methods to analyze the microbial flora of root-filled teeth with periradicular lesions. Twenty-one samples from previously root-filled teeth were collected from patients with periradicular lesions. Microorganisms were cultivated, isolated and biochemically identified. In addition, ribosomal DNA of bacteria, fungi and archaea derived from the same samples was amplified and the PCR products were used to construct clone libraries. DNA of selected clones was sequenced and microbial species were identified, comparing the sequences with public databases. Microorganisms were found in 12 samples with culture-dependent and -independent methods combined. The number of bacterial species ranged from 1 to 12 in one sample. The majority of the 26 taxa belonged to the phylum Firmicutes (14 taxa), followed by Actinobacteria, Proteobacteria and Bacteroidetes. One sample was positive for fungi, and archaea could not be detected. The results obtained with both methods differed. The cloning technique detected several as-yet-uncultivated taxa. Using a combination of both methods 13 taxa were detected that had not been found in root-filled teeth so far. *Enterococcus faecalis* was only detected in two samples using culture methods. Combining the culture-dependent and –independent approaches revealed new candidate endodontic pathogens and a high diversity of the microbial flora in root-filled teeth with periradicular lesions. Both methods yielded differing results, emphasizing the benefit of combined methods for the detection of the actual microbial diversity in apical periodontitis.

## Introduction

Endodontic failures correspond with a persistence of periradicular lesions [1], [2]. To conserve the tooth a revision of the endodontic treatment becomes necessary, because otherwise persistent microorganisms or secondary infections mainly caused by insufficient coronal restoration can lead to loss of the tooth. Microorganisms have been isolated in 35–100% of root-filled teeth with periradicular lesions [3], [4], [5], [6], [7], [8], [9], [10], [11], [12].

Earlier studies using culture methods have revealed a distinctly different microbial flora compared to primary infections, including mostly gram-positive bacteria, predominantly facultative anaerobes and few obligate anaerobes [4], [13]. However, solely applying culture methods can lead to an underestimation of as-yet-uncultivated species, considering that estimates suggest that only approximately 40 to 50% of the bacteria present in the oral cavity can be cultivated [14] With the molecular techniques of culture-independent open-ended analysis of 16S rRNA genes it became possible to detect uncultivated bacteria or uncultivable biotypes of known species [15] allowing for investigation of the actual microbial diversity of infected root canals. To date, only three culture-independent studies using the 16S-rDNA cloning technique have been done to analyze the microbial diversity of secondary endodontic infections [8], [16], [17]. Yet PCR and cloning analysis will also lead to some bias due to the detection of DNA from dead cells, differential DNA-extraction or preferential DNA amplification [18]. This might cause an overestimation of the role of certain species that can reach the root canal but might not actively grow there [19].

A better insight into the composition of the microbial flora of treated root canals is essential to further our understanding of the etiology of apical lesions and to improve treatment strategies for bacterial apical periodontitis which seek to eradicate the microorganisms present [20]. Our study aimed to investigate the microbial flora of treated root canals associated with apical periodontitis combining cultural methods with the culture-independent approach. The culture-independent method used was 16S rRNA clone library analysis. This combined approach applied to a fairly large sample set should be able to better characterize the local microbial communities which until now has not been satisfactorily done [21].

## Materials and Methods

### Clinical Material

Twenty-one patients who had been referred to the University Clinic and Dental Hospital, University of Freiburg, for endodontic retreatment participated in this study. All of them gave their written informed consent to the study protocol, which had been approved by the ethics committee (Nr. 140/09, University of Freiburg). Patients with conditions that met the following criteria were excluded from the study: 1) severe systemic disease, 2) poor tooth prognosis and improvement of initial condition unlikely, 3) pregnancy or lactation, 4) use of antibiotics within the last 30 days, 5) participation in any other clinical study within the last 30 days.

Endodontic treatment of all teeth had been completed at least 2 years earlier and all teeth exhibited apical periodontitis in the radiographic examination. In all cases retreatment was indicated and previous root canal treatment considered a failure. No direct exposure of the root canal filling material to the oral cavity was evident. All teeth were asymptomatic. Teeth with obturation material that did not reach within 4 mm of the radiographic apex or could not be isolated with a rubber dam were excluded from the study.

### Sampling Procedure

All samples were collected under strictly aseptic conditions. Samples for bacterial growth were transferred into vials containing 0.75 ml reduced transport fluid (RTF) [22] and stored at −80°C. The sampling procedure was conducted as described in earlier studies in detail [11], [12]. In brief, the tooth and surrounding field were cleaned with 30% hydrogen peroxide (H_2_O_2_) and swabbed with a 2.5% sodium hypochlorite solution (NaOCl). Endodontic access was achieved with a sterile high-speed carbide bur until the root filling was exposed. Then the tooth and the adjacent rubber dam were disinfected a second time using 30% hydrogen peroxide (H_2_O_2_) and 2.5% sodium hypochlorite solution (NaOCl). The cavity was swabbed with 5% sodium thiosulfate solution to inactivate the NaOCl. To assess efficacy of the disinfection, a sterile foam pellet was moistened in sterile 0.9% NaCl solution and used to swab the access cavity and the tooth surface. If bacterial growth occurred in these quality control samples, the tooth was excluded from the study.

Coronal gutta-percha was removed with Gates-Glidden drills. The working length was established radiographically and with the aid of an electronic apex locator (Raypex 5; VDW, Munich, Germany). The canal was enlarged from 0.5 to 2 mm from the radiographic apex with a minimum ISO size 35 nickel-titanium K-type file. Teeth that could not be filed to this length were excluded from the study. No solvent was used at any time. After introducing approximately 40 µl sterile saline solution (0.9% NaCl) into the canal with a sterile syringe, three sequential sterile paper points of type ISO 25, taper 04 (ROEKO, Langenau, Germany) were placed into the working length to soak up the fluid. Each paper point was kept inside the canal for 1 minute and then transferred into a sterile vial containing RTF. Finally, conventional retreatment was finished after root canal disinfection, and the root canal was filled by using vertical compaction.

### Cultural Analysis of the Microflora

The culture method was performed as described elsewhere [12]. The vials containing the samples in RTF were thawed at 36°C in a water bath and vortexed for 30–45 seconds. To isolate and identify the microorganisms, 350 µl of the undiluted sample (corresponding to a dilution of 10^−2^ of the original root canal bacteria sampled with paper points) and serial dilutions thereof were cultivated. Serial dilutions (10^−1^ to 10^−3^) were prepared in peptone yeast medium (PY) containing cysteine hydrochloride [23]. Each dilution was plated on yeast-cysteine blood agar plates (HCB), on Columbia blood agar plates (CBA) and on bile esculin plates. HCB agar plates were used to cultivate anaerobic bacteria at 37°C for 10 days (anaerobic chamber, GENbox BioMérieux® sa, Marcy l’Etoile-France). CBA agar plates were incubated at 37°C and 5%–10% CO_2_ atmosphere for 5 days to cultivate aerobic and facultative anaerobic bacteria. Bile esculin agar plates were used to cultivate *Enterococcus faecalis* at 37°C and 5%–10% CO_2_ atmosphere for 2 days. Colony types were noted and counted to calculate the number of colony forming units (CFU) per ml in the original sample. All colony types were sub-cultivated to obtain pure cultures.

Gram stains were prepared and bacterial cell morphology was determined using light microscopy (Axioscope; Zeiss, Jena, Germany; 1000× magnification). The biochemical identification of anaerobic microorganisms was performed by routine anaerobic methods, including commercial tests (rapid ID 32 A; Bio Merieux, Marcy-l’Etoile, France; rapid ANA II; Innovativ Diagnostic Systems, Innogenetics, Heiden, Germany). Both tests use conventional and chromogenic substrates for differentiation, and were performed according to the manufacturers’ instructions. To identify the aerobic and facultative anaerobic microorganisms, biochemical characteristics were analyzed with commercially available tablets (Rosco Diagnostics, Taastrup, Denmark) and API 20 Strep (Bio Merieux). All tests were performed according to the manufacturers’ instructions. Isolates that could not be identified using the above mentioned methods were analyzed by Maldi-TOF (Maldi Biotyper, Bruker Daltonik GmbH, Bremen, Germany) and with universal bacterial PCR with the following Primers: TP16U1: 5′-AGAGTTTGATCMTGGCTCAG-3′ and RT16U6: 5′-ATTGTAGCACGTGTGTNCCCC-3′ followed by sequencing. Sequencing was performed on a 3130 Genetic Analyzer (Applied Biosystems, Life Technologies GmbH, Darmstadt, Germany).

### DNA-Isolation

After removal of 350 µl of the samples in RTF, the remainder was used to isolate bacterial and fungal DNA. In preliminary experiments we had tested 3 different kits, designed particularly for DNA extraction from small samples, and different DNA extraction procedures to ensure the best possible yield and sensitivity for our protocol. Samples were centrifuged at 16.000 g for 10 min and the supernatant was discarded. Lysis of microbial cells was performed using a Precellys 24 bead mill homogenizer (PEQLab Biotechnologie GmbH, Erlangen) in ATL buffer (QiaAMP Micro Kit; Qiagen, Hilden, Germany). The vials were shaken twice at 3500 rpm for 30 s. The DNA was subsequently purified with the QiaAMP Micro Kit (Qiagen, Hilden, Germany) according to the manufacturer’s protocol for tissue samples. The total microbial DNA was eluted twice with 50 µl AE buffer (Qiagen) and then stored at −20°C.

### PCR Amplification of 16S and 18S rRNA Genes

Bacterial and archeal 16S and fungal 18S rRNA genes were amplified using the following universal primers, which have been previously published.

The bacterial primers used were 27F-YM (5′-AGAGTTTGATYMTGGCTCAG-3′) and 1492R (reverse: 5′-TACGGYTACCTTGTTACGACTT-3′) [24], [25], the primers for archaea were A109F (forward: 5′-ACKGCTCAGTAACACGT-3′) and A934R (reverse: 5′-GTGCTCCCCCGCCAATTCCT-3′) [26]. Fungal 18S-rRNA coding genes were amplified with ITS1-F (forward: 5′- CTTGGTCATTTAGAGGAAGTAA-3′) and ITS4-R (reverse: 5′- TCCTCCGCTTATTGATATGC-3′) [27]. The PCR amplification was performed in a total volume of 50 µl. The reaction mixture contained 1× PCR buffer (Qiagen), 0.2 mM each of the four deoxyribonucleoside triphosphates (dNTPs; PEQLAB, Erlangen, Germany), 0.5 µM of forward and reverse primers, 2 U Taq-Polymerase (Qiagen) and 5 µl of the isolated sample DNA. The PCR cycling conditions consisted of an initial denaturation step at 94°C for 2 min, followed by 35 cycles with denaturation at 94°C for 1 min, annealing at 55°C for 1 min and extension at 72°C for 1.5 min, with a final extension step at 72°C for 10 min. A no-template control and a positive control were included in each set of PCR reactions. PCR reaction products were analyzed by electrophoresis in a 1.5% agarose gel and positive reactions were used to prepare clone libraries.

### Cloning of PCR Products and Analysis of Clone Libraries

The 16S-rDNA and 18S-rDNA amplification products were ligated into the pCR®2.1-TOPO® plasmid vector using the TOPO TA Cloning® Kit (Invitrogen, Life Technologies, Darmstadt, Germany) according to the manufacturer’s protocol. All white clones from each library were picked and the presence of inserts was confirmed by PCR amplification with their respective primers followed by gel electrophoresis. PCR products of all recombinants were subjected to a restriction enzyme digest with the following restriction endonucleases: PCR products of recombinants that resulted from the universal bacterial PCR were digested with Hha I, Rsa I and Hinf I (New England Biolabs GmbH, Frankfurt, Germany), while those from the universal fungal PCR were digested with Hha I and Alu I (New England Biolabs). Fragment length patterns were compared and grouped if they were identical. One representative clone was selected from each group and used for sequencing.

The selected clones with inserts of the correct size were grown in Luria-Bertani liquid medium with kanamycin (50 mg/ml) at 37°C overnight. Plasmid DNA extraction was then prepared using the PureLink Quick Miniprep Kit (Invitrogen, Life Technologies, Darmstadt, Germany). Sequencing was performed on an automated ABI 3730×l DNA Analyzer (Applied Biosystems, Life Technologies GmbH, Darmstadt, Germany).

### Sequence Analysis

The sequence data obtained from the ABI sequencer was visually proofread and edited using the Ridom TraceEdit software (Ridom GmbH Münster, Germany). The partial and almost full-length 16S- or 18S-rDNA sequences were compared to those from public sequence databases, Genbank, EMBL and DDBJ using the BLAST program. The program was run through the server hosted by the National Center for Biotechnology Information (http://www.ncbi.nigh.gov/BLAST) [28], [29]. Sequences that showed 98% similarity or less with public database sequences were checked for chimeras with the Pintail program, version 1.0 [30]. Chimeric sequences were excluded from further analysis. If no chimeras were detected, bidirectional sequencing was done, trimmed sequences were assembled using BioEdit [31] and another “blastn” search was run. Sequences with a 99–100% match to a database sequence were considered to be of the same species as the one with the highest similarity and score bits.

Additionally, all 16S-rDNA sequences were compared with the database sequences of the Ribosomal Database Project (http://rdp.cme.msu.edu/) [32] and the Human Oral Microbiome Database (HOMD, http://www.homd.org/) [33] to confirm the results of the “blastn” search and to obtain further information. Sequences that could not be assigned to any database sequence were considered to be a novel phylotype if they were less than 98% similar to the closest Genbank entry.

The 16s-rDNA sequences obtained were used for further comparative sequence analysis and phylogenetic analysis using the tools implemented in the software package ARB [34]. To implement the obtained sequences the reference dataset LTPs 106_SSU from the SILVA project [35] was used. Alignments were performed using the SINA Aligner plugin. After manual correction of the alignment, a phylogenetic tree was constructed with the ARB Neighbour joining method applying the Felsenstein correction and bootstrapping was calculated based on 500 replicates. Partial sequences were added without allowing changes of the tree topology by use of the ARB “parsimony interactive” method.

## Results

### Cultural Method

A total of 21 samples were analyzed using the cultural method. One tooth had to be excluded from further analysis because of bacterial contamination of the quality control sample. The results of the culture findings are shown in Table 1.

**Table 1:** Comparison of microorganisms in root-filled teeth with periradicular lesions using cultural methods and 16S-rDNA clone library analysis.

Sample	Cultural method	16S r DNA cloning technique
1R	*Enterococcus faecalis*	negative
2R	negative	*Enterococcus gallinarum/casseliflavus, Candida parapsilosis*
3R	*Parvimonas micra*	negative
4R	negative	negative
5R	negative	*Lactobacillus gasseri*
6R	negative	negative
7R	*Proteus hauseri/vulgaris, Streptococcus oralis, S. salivarius, Lactobacillus* *fermentum, Actinomyces oris, Neisseria elongata, Dialister invisus*	*Proteus hauseri/vulgaris, Streptococcus mutans, Peptostreptococcus* *stomatis, Selenomonas sp., Olsenella profusa, Delftia sp.*
8R	negative	negative
9R	negative	*Streptococcus* sp.
10R	negative	negative
11R	*Enterococcus faecalis*	*Exiguobacterium aurantiacum, Pantoea agglomerans*
12R	negative	*Uncultured Neisseria clone*
13R	negative	*Phocaeicola abscessus*
14R	negative	negative
15R	*Streptococcus mutans, S. parasanguinis, Propionibacterium acnes,* *Rothia dentocariosa*	negative
16R	excluded	excluded
17R	negative	negative
18R	*Corynebacterium minutissimum*	negative
19R	negative	negative
20R	negative	negative
21R	*Rummeliibacillus stabekisii, Propionibacterium acnes*	negative

Seven teeth harbored cultivable microorganisms in the root canal sample. The density of microorganisms ranged from 1×10^3^ CFU/ml to 6.8×10^4^ CFU/ml for aerobic cultivation (with a median of 3×10^3^ CFU/ml) and from 1×10^3^ CFU/ml to 2.4×10^4^ CFU/ml for anaerobic cultivation (with a median of 2.5×1×10^3^ CFU/ml). Overall, 14 different bacterial species were identified; fungi could not be isolated from any of the samples. The number of species obtained using the culture method alone varied from 1 to 7 in one sample.

Three taxa were found several times; *Enterococcus faecalis, Streptococcus* spp. and *Propionibacterium acnes* were each present in 2 different samples. The species isolated using standard culture methods belonged to the phyla Firmicutes, Actinobacteria and Proteobacteria, with the largest percentage belonging to the Firmicutes (15 Taxa). Most isolated species were either aerobic or facultative anaerobic organisms, only 4 belonged to strictly anaerobic genera. A total of five taxa, *Neisseria elongata*, *Actinomyces oris*, *Corynebacterium minutissimum*, *Proteus hauseri/vulgaris* and the genus *Rummeliibacillus* were detected for the first time in filled root canals.

### Analysis of 16S-rDNA Clone Libraries and Comparison with the Culture Method

The universal bacterial PCR performed on the 21 DNA samples (followed by construction of clone libraries) showed positive results for 7 samples and the universal fungal PCR for one sample. Archeal 16S-rDNA sequences could not be amplified from any of the samples. One tooth had to be excluded from further analysis since the quality control showed a positive result (see above). The microorganisms identified after sequencing of 58 clones are listed in Table 2. Alternative sequences are stated in the table, where identity scores between the analyzed clone and the top two public database sequences were the same or very close. Of the 14 different taxa that were found, most belonged to the phylum Firmicutes (7 taxa), some to the phyla Proteobacteria (4 taxa), Actinobacteria (1 taxon) and Bacteroidetes (1 taxon), while one sample harboured a fungal species. The majority of identified species were aerobic or facultative anaerobic organisms, only 4 were obligate anaerobic organisms. Only members of the genus *Streptococcus* were found in more than one case and 4 of the taxa found represented as yet uncultivated taxa. The number of taxa per sample detected by molecular analysis alone ranged from 1 to 6.

**Table 2:** Bacterial taxa found in clinical samples of root-canal treated teeth with apical periodontitis with 16S-rDNA cloning technique.

Sample	Clone^a)^	Bacterial Taxa^b)^	% Identity^c)^
		Bacteria	
2R	56	*Enterococcus gallinarum.* [HQ378521], *Enterococcus casseliflavus* [EU151766]	98
5R	51	*Lactobacillus gasseri* [AF243156]	99
7R	179	*Streptococcus mutans* [AE014133]	100
7R	199	*Selenomonas* sp. *oral clone* [AF287794]	99
7R	218	*Peptostreptococcus stomatis* [GU401283]	98
7R	197	*Olsenella profusa* [NR_036821]	99
7R	215	*Proteus hauseri* [AB594762] oder *vulgaris* [NR_025336]	99
7R	171	*Uncultured Delftia* sp. [GU563748]	99
9R	111	*Streptococcus* sp. [AF316595]	99
11R	85	*Exiguobacterium aurantiacum* [JN644574]	99
11R	90	*Pantoea agglomerans* [EU304255.1]	99
12R	7	*Uncult. Neisseria cl.* [EU794238]	99
13R	45	*Phocaeicola abscessus* [AB595138]	99
		Fungi	
2R	265	*Candida parapsilosis* [GQ395610]	99

Accession numbers are given in brackets.

a) Only one clone name is given as an example if sequences were detected in several clones.

b) Match for sequenced almost full length and partial 16S rRNA-genes from clones from 21 cases; accession numbers are shown in brackets.

b) Results are based on BLAST similarity scores for cloned sequences (800–1500 bp).

**Figure 1:**
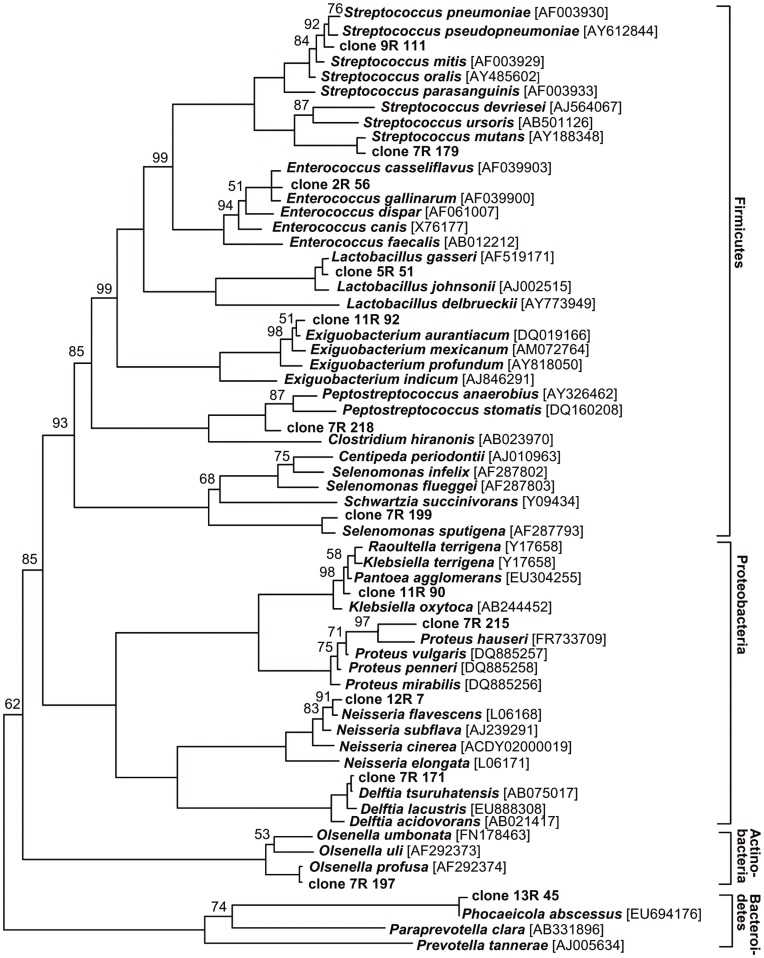
Phylogenetic analysis of bacterial taxa found in clinical samples of root-canal treated teeth with apical periodontitis. 16S-rDNA gene sequences were aligned using the SINA plugin (ARB software package) and distances were calculated using the Neighbour-joining method with Felsenstein correction. Bootstrap values over 50% (based on 500 replicates) are shown on nodes. The scale bar indicates 5% sequence divergence.

Of all identified taxa, 8 were found in secondary/persistent root canal infections for the first time: *Enterococcus gallinarum/casseliflavus*, *Lactobacillus gasseri*, *Olsenella profusa*, *Proteus hauseri/vulgaris*, *Exiguobacterium aurantiacum*, *Phocaeicola abscessus*, *Pantoea agglomerans*, *Delftia* spp. had not been detected in root-filled teeth before. 4 clone sequences had high percentage identities with database sequences that were only identified to the genus level (*Selenomonas* sp., *Streptococcus* sp., *Delftia* sp. and *Neisseria* sp.); these clones belonged to as yet uncultivated phylotypes. Figure 1 shows the phylogenetic analysis of all taxa found with the 16S-rDNA cloning technique. Sequences of identified clones will be deposited in the GenBank database.

In comparison to the culture analysis, the same amount of samples was positive using the 16S-rDNA cloning technique (7). With this technique microorganisms were found in 5 samples which were negative for the culture analysis, yet bacteria were also isolated from 5 samples that were negative with the 16S-rDNA cloning technique. Both methods combined revealed microorganisms in 12 of the 21 root-filled teeth. The number of taxa per sample that were detected with both methods combined ranged from 1 to 12. In 6 cases a single taxon was present (*Parvimonas micra*, *Lactobacillus gasseri*, *Streptococcus* sp., *Phocaeicola abscessus*, *Neisseria* sp. *clone* and *Corynebacterium minutissimum*) and in 3 cases 2 species were present (*Streptococcus* sp. and *Enterococcus faecalis*; *Enterococcus gallinarum*/*casseliflavus* and *Candida parapsilosis*; *Propionibacterium acnes* and *Rummeliibacillus stabekisii*). One case each revealed polymicrobial infections with 3, 4 and 12 species resp. (*Exiguobacterium aurantiacum*, *Pantoea agglomerans* and *Enterococcus faecalis*; *Streptococcus parasanguis*, *Streptococcus mutans*, *Rothia dentocariosa*, and *Propionibacterium acnes*; *Streptococcus mutans*, *Streptococcus oralis*, *Streptococcus salivarius*, *Lactobacillus fermentum*, *Actinomyces oris*, *Proteus hauseri/vulgaris*, *Neisseria elongate*, *Dialister invisus*, *Selenomonas* sp., *Peptostreptococcus stomatis*, *Olsenella profusa* and *Delftia* sp.).

Fourteen different taxa were identified using both the culture analysis and 16S-rDNA cloning technique respectively, but they were not the identical taxa except for one species that was identified with both methods in the same sample (*Proteus vulgaris/hauseri* in sample Nr. 7). In addition, one species, *Streptococcus mutans* was identified with both methods but in different samples. Therefore, a total of 26 different taxa were identified with both methods. The diversity of the microbial flora detected with both methods was very similar with regard to the phyla of the microorganisms present in the samples. However, only the 16S-rDNA cloning method revealed 1 fungal species and 1 species of the phylum Bacteroidetes.

## Discussion

The microflora of root-filled teeth with periapical lesions have been primarily studied by culture dependent methods, as well as by species-specific PCR. Only three studies analysed microorganisms of secondary endodontic infections by a culture-independent 16S-rDNA cloning approach [8], [16], [17]. Furthermore, no study to date has shown a direct comparison of both methods with an adequate number of clinical samples [8]. The present study attempts to fill this gap in examining the microflora of root-filled teeth applying the two approaches in parallel. This comparison revealed a high diversity of microorganisms and a very high inter-individual variability in the composition of the flora, with both methods showing differing results and complementing each other. Both methods revealed several taxa that had not been reported in treated root canals. However, only with the cloning technique a number of taxa was found that belonged to as-yet-uncultivated genera/species.

Previous findings revealed bacterial colonization in treated root canals in 44% to 85% of the samples with culture methods [10], [36] and in 65% to 100% of the samples with molecular analysis [8], [11], [16]. The 33% positive samples found with the cultural method in this study and the percentage of positive samples found with molecular analysis (33%) is lower than in earlier studies (although the two methods combined revealed microorganisms in 57% of the samples). On the one hand, not every case of diagnosed apical periodontitis is necessarily associated with microbial infection [5], and on the other hand several reasons could be responsible for the inability to detect present microorganisms in some cases with neither of the methods. Microorganisms might be present in such low numbers that they escaped detection or even sampling due to inaccessibility of certain areas of the root canal or to removal of microorganisms adhered to filling material and debris [10]. All samples were split in half for the parallel analysis with both methods, which might have lowered the concentration of some species to under the detection limit. Other reasons are inherent to the particular methods. The molecular analysis was able to reveal yet uncultivated and fastidious species which are not detected by culture methods. Yet there is also a certain bias introduced with the molecular analysis. Even though the DNA extraction method was very effective, DNA from strains present in low abundance can be lost. Differential amplification of 16S-rDNA sequences as a result of varying concentrations of cells and rrn ribosomal operon copy numbers might have had an additional effect [8], [18]. Considering the low viable counts of the bacteria isolated in this study (10^3^ CFU/ml–10^4^ CFU/ml), these results indicate that the overall abundance of microorganisms in the samples was in fact quite low (close to detection limit for both methods). In addition, the specific sample selection of asymptomatic teeth only presumably affected the results obtained in regard to the concentration of bacterial cells. Earlier studies [11], [37] reported on the high variation in prevalence of microorganisms in root-filled teeth. Thus these might be due to inclusion of high numbers of teeth with persistent symptoms or a history of symptoms in some studies whereas other studies only included asymptomatic teeth showing radiographic evidence of periradicular lesions.

To date there is still no sufficient knowledge on the microorganisms involved, and especially the role of *Enterococcus faecalis* is still discussed contradictorily and no consensus is reached yet. Additionally, the role of fungi and methanogenic bacteria in asymptomatic endodontic infections is still unclear. On the other hand studies have shown that for the most part symptomatic teeth resemble primary infections concerning their microbial profile [13] which is already quite well understood.

The high prevalence of microorganisms in earlier reports has also been explained by their inclusion of teeth with a very low quality of the previous root filling [4], [16], [11], [38]. The number of species found per sample in this study ranged from 1 to 4 for most samples except for one harbouring 12 different species. This confirms previous statements that the quality of the initial root canal filling corresponded to the number of species isolated and the bacterial density [13], [6], [4]. Well treated canals revealed about 1–3 species whereas canals with very poor treatment revealed up to 30, similar to untreated canals with necrotic pulp.

There is significant evidence for an endogenous path of infection and it is undisputed that secondary endodontic infection can occur e.g. via coronal leakage if the primary filling is inadequate. Various microorganisms that are part of the dental plaque can obtain access to the root canal and persist therein [1], [48].

Previous studies from our group have been able to show that endodontic and salivary isolates of *Enterococcus faecalis* are able to integrate and persist in oral biofilm [39]. Further research comparing the genotype of *E. faecalis* isolates provided evidence that foodborne *E. faecalis* isolates could integrate in oral biofilm *in situ* [40]. Therefore it can be possible for *E. faecalis* e.g. originating from cheese or other foods to act as a causative agent of secondary root canal infections. From these experiments we can conclude that exogenous as well as endogenous infections are likely.

The overall diversity of the microbial flora detected with culture methods was similar to the findings with the cloning technique in that gram-positive and facultative anaerobic bacteria dominated. These results are in agreement with previous studies reporting that the majority of species found in treated canals belonged to the phylum Firmicutes, followed by Actinobacteria and Proteobacteria (s. Table 3). In contrast, primary infections have revealed many more representatives of Bacteroidetes, followed by Firmicutes as well as more obligate anaerobes [13], [41]. Some of the species found in this study, e.g. *Dialister invisus* or *Parvimonas micra* are found very frequently in primary cases [13], suggesting that these bacteria might have survived primary endodontic treatment, resulting in a persistent infection.

**Table 3:** Comparison of microbial profiles of root-filled teeth with periradiular lesions found in studies by culture-dependent and/or -independent approaches.

Reference	Method of isolation and identification	Taxa found with culture-dependant methods*	Taxa found with culture-independent methods*
Molander et al. 1998 [5]	Culture, morphology, biochemistry	*Enterococcus* spp., *Eubacterium alactolyticum*, *Streptococcus* spp, *Staphylococcus epidermidis*, *Lactobacillus* spp., *Actinomyces* spp., *Bacillus* sp., *Peptostreptococcus* spp., *Veillonella* sp., *Propionibacterium* spp., *Escherichia coli*, *Citrobacter freundii*, *Klebsiella* spp., *Enterobacter agglomerans*, *Proteus* sp., *Pseudomonas* sp., *Wolinella* sp., *Prevotella* sp., *Fusobacterium* spp., *Candida albicans*	
Sundqvist et al. 1998 [10]	Culture, morphology, biochemistry	*Enterococcus faecalis*, *Streptococcus anginosus*, *Streptococcus constellatus*, *Streptococcus intermedius*, *Streptococcus mitis*, *Streptococcus parasanguis*, *Peptostreptococcus micros*, *Lactobacillus catenaforme*, *Pseudoramibacter alactolyticus* ( = formerly: *Eubacterium* alactolyticum), *Eubacterium timidum*, *Propionibacterium acnes*, *Propionibacterium propionicum*, *Actinomyces israelii*, *Bacteroides gracilis*, *Fusobacterium nucleatum*, *Candida albicans*.	
Hancock et al. 2001 [38]	Culture, morphology, biochemistry	*Enterococcus* spp., *Streptococcus epidermidis*, *Staphylococcus aureus*, *Lactobacillus* spp., *Bacillus* spp., *Peptostreptococcus* spp., *Eubacterium* spp., *Veillonella intermedia*, *Actinomyces* spp., *Corynebacterium* spp., *Eikenella corrodens*, *Prevotella intermedia*, *Porphyromonas* spp., *Fusobacteriuim* spp., *Candida albicans*	
Cheung et al 2001 [4]	Culture, morphology, biochemistry	*Streptococcus constellatus, S. mitis, S. mutans, Staphylococcus spp., Gemella morbillorum, Peptostreptococcus spp., Eubacterium lentum, Veillonella spp., Propionibacterium propionicum, Klebsiella oxytoca, Enterobacter cloacae, Proteus mirabilis, Serratia spp., Pseudomonas spp., P. aeruginosa, Neisseria spp., Campylobacter spp., Prevotella prevotii, P. saccharolyticus, Porphyromonas asaccharolytica, C. albicans*	
Peculiene 2001 [6]	Culture, morphology, biochemistry	*Enterococcus faecalis, Actinomyces viscosus, Escherichia coli, Klebsiella pneumoniae, Proteus mirabilis, Fusobacterium nucleatum, Candida albicans*	
Rolph et al. 2001 [8]	Culture, morphology, biochemistry, 16S rDNA cloning method	*Streptococcus intermedius, S. mitis, S. oralis, Peptostreptococcus micros, Eubacterium* spp., *Eubacterium spp., Peptostreptococcus micros, Peptostreptococcus prevotii, Propionibacterium acnes, Propionibacterium granulosum, Veillonella* spp., *Actinomyces israelii, A. naeslundii, A. viscosus, P. prevotii Porphyromans endodontalis*	*Enterococcus faecalis, Streptococcus anginosus, S. gordonii, S. intermedius strain* VAMC3276, Uncultured *S. mitis, S. salivarius, S. sanguis-like oral clone AP60-3, Gemella haemolysans, Lactobacillus spp., L. casei, L. fermentum, L. paracasei, Micrococcus* strain MC6, *Peptostreptococcus* sp., *Selenomonas infelix, Selenomonas* sp. oral clone CS015, *Dialister* sp. oral clone BS095, *Eubacterium sp.* oral strain A35MT, *E. infirmum* W 1471, *E. brachy, E. yurii, Veillonella* sp., *V. dispar Mogibacterium* sp., *Solobacterium moorei, Fusobacterium naviforme, Propionibacterium acnes, Rothia dentocarios, Pantoea sp., Capnocytophaga gingivalis, Cytophaga* sp. strain P1, *Prevotella* sp., *P. nigrescens, P. oris, F. nucleatum*
Pinheiro et al 2003 [36]	Culture, morphology, biochemistry	*Enterococcus faecalis, E. faecium, Streptococcus anginosus, S. constellatus, S. mitis, S. mutans, S. oralis, S. sanguis, S. salivarius, Lactobacillus acidophilus, Lactococcus lactis, Staphylococcus lentus, Peptostreptococcus prevotii, P. micros, P. magnus, P. saccharolyticus, Eubacterium lentum, Gemella morbillorum, Veillonella spp., Actinomyces naeslundii, A. odontolyticus, A. viscosus, Bifidobacterium* spp., *Clostridium subterminale Propionibacterium acnes, P. propionicum, Haemophilus aphrophilus, Capnocytophaga* spp., *Prevotella buccae, P. corporis, P. loescheii, P. melaninogenica, P. nigrescens, Fusobacterium necrophorum, F. nucleatum, Candida albicans*	
Pinheiro et al. 2003 [62]	Culture, morphology, biochemistry	*Enterococcus faecalis, Streptococcus anginosus, S. constellatus, S. sanguis, S. mitis, S. oralis, S. salivarius, Lactobacillus acidophilus, Staphylococcus aureus, S. lentus Peptostreptococcus prevotii, P. micros, P. magnus, P. saccharolyticus, Gemella morbillorum, Propionibacterium acnes, P. propionicum, Veillonella spp., Actinomyces naeslundii, A. odontolyticus, A. viscosus, Haemophilus aphrophilusm, Capnocytophaga* spp., *Prevotella buccae, P. corporis, P. nigrescens, P. loescheii, P. melaninogenica, Fusobacterium necrophorum, Candida albicans*	
Gomes et al. 2004 [37]	Culture, morphology, biochemistry	*Enterococcus faecalis, Streptococcus anginosus, S constellatus, S. mitis, S. salivarius, S. sanguis, Lactobactillus acidophilus, Staphylococcus lentus, Gemella morbillorum, Peptostrepto-coccus prevotii, P. magnus,P. micros, P. saccharolyticus, Actinomyces naeslundii, Propionibacterium acnes, Prevotella buccae, P. denticola, P. intermedia/nigrescens, P. loescheii, P. melaninogenica, Porphyromonas gingivalis, Fusobacterium necrophorum*	
Rôças et al. 2004 [7]	Species-specific PCR		*Enterococcus faecalis, Streptococcus sp., Tannerella forsythensis*
Siqueira and Rôças 2004 [9]	Species-specific PCR	*Enterococcus faecalis, Streptococcus spp., Peptostreptococcus micros, Pseudoramibacter alactolyticus, Dialister pneumosintes, Filifactor alocis, A radicidentis, Propionibacterium propionicum, Treponema denticola, Campylobacter gracilis, C. rectus, Prevotella intermedia, Tannerella forsythensis, Porphyromonas gingivalis, P. endodontalis, Fusobacterium nucleatum, Candida albicans*	
Siqueira and Rôças 2005 [69]	Species-specific PCR		*Dialister invisus, Olsenella uli, Synergistes oral clone*
Gomes et al. 2005 [70]	Culture, morphology, biochemistry, species-specific PCR	*Prevotella intermedia, P. nigrescens*	*Prevotella intermedia, P. nigrescens, Porphyromonas gingivalis, P. endodontalis*
Peciuliene 2000 [64]	Culture, morphology, biochemistry	*Enterococcus faecalis*	
Kaufman et al. 2005 [63] Rôças 2004 [68]	Genus-specific PCR, DNA-sequencing; species-specific PCR		*Enterococcus faecalis*
Sedgley et al. 2006 [52], Gomes et al 2006 [65], Zoletti et al. 2006 [67]	Culture, morphology, biochemistry; species-specific PCR, Real-time PCR	*Enterococcus faecalis*	*Enterococcus faecalis*
Gomes et al 2008 [66]	species-specific PCR		*Enterococcus faecalis, Peptostreptococcus micros, Filifactor alocis, Treponema denticola, Prevotella denticola P. intermedia, P. nigrescens, Tannerella forsythensis, Porphyromonas gingivalis, P. endodontalis*
Sakamoto et al. 2008 [16]	16S rDNA cloning method		*Enterococcus sp., E. faecalis, Streptococcus sp. S. constellatus, S. mutans, S. oralis, S. pyogenes, S. sanguinis, Peptostreptococcus sp., P. stomatis, Pseudoramibacter alactolyticus, Eubacterium sp., E. yurii, Lachnospiraceae oral clone, Dialister sp., D. invisus, Clostridiales oral clone, Veillonella sp., Selenomonas sp., Shuttleworthia satelles, Solobacterium sp., Actinomyces sp. A. naeslundii, A. radingae, Atopobium rimae, Bifidobacterium sp Corynebacterium sp., C. durum, Olsenella genomsp O. uli, Propionibacterium sp., Brevundimonas diminuta, Burkholderiales oral clone Campylobacter showae, Enterobacteriaceae oral clone, Dechlorospirillum sp. DB Escherichia sp. oral clone, Mesorhizobium amorphae, Paracoccus sp. oral clone, Petrobacter succinimandens, Pseudomonas sp. P. aeruginosa, P. putida, Stenotrophomonas maltophilia, Terrahaemophilus aromatici-vorans, Bacteroidales oral clone, Flavobacteriaceae genomosp., Prevotella baroniae, P. denticola, P. nigrescens, P. oris, Porphyromonas gingivalis, Tannerella sp., T. forsythia, Fusobacterium sp., F. nucleatum, Synergistes sp.*
Schirrmeister 2009 [12]	Culture, morphology, biochemistry, universal bacterial PCR and DNA sequencing	*Enterococcus avium*, *E. faecalis*, *E. faecium*, *Streptococcus anginosus*, *S. intermedius*, *Streptococcus*, *Vagococcus fluvialis* isolate EH-Endo, *Staphylococcus epidermidis, Dialister invisus*, *Megasphaera* spp., *Parvimonas micra*,, *Slackia exigua*, *Solobacterium moorei*, *Veillonella parvula*, *Actinomyces giorgiae*, *Atopobium rimae*, *Olsenella uli*, *Propionibacterium acnes*, *Campylobacter gracilis*, *Enterobacter amnigenus*, *Klebsiella pneumoniae*, *Tannerella forsythia*, *Porphyromonas gingivali*s, *Fusobacterium nucleatum*, *Synergistes* spp.	*Enterococcus avium*, *E. faecalis*, *E. faecium*, *Streptococcus anginosus*, *S. intermedius*, *Streptococcus*, *Vagococcus fluvialis* isolate EH-Endo, *Staphylococcus epidermidis, Dialister invisus*, *Megasphaera* spp., *Parvimonas micra*,, *Slackia exigua*, *Solobacterium moorei*, *Veillonella parvula*, *Actinomyces giorgiae*, *Atopobium rimae*, *Olsenella uli*, *Propionibacterium acnes*, *Campylobacter gracilis*, *Enterobacter amnigenus*, *Klebsiella pneumoniae*, *Tannerella forsythia*, *Porphyromonas gingivalis, Fusobacterium nucleatum*, *Synergistes* spp.
Rôças and Siqeira 2012 [71]	Reverse capture checkerboard assay		*Enterococcus faecalis, Streptococcus spp., Parvimonas micra, Peptostreptococcus stomatis, Dialister invisus, D. pneumosintes, Filifactor alocis, Pseudoramibacter alactolyticus, Selenomonas sputigena, Solobacterium moorei, Actinomyces israelii, Atopobium genomospecies* C1, *A. rimae Olsenella uli, Propionibacterium acidifaciens, P. acnes, Treponema denticola, T. socranskii, Eikenella corrodens, Campylobacter rectus, Pseudomonas aeruginosa, Bacteriodetes clone* X083, *Prevotella baroniae, Porphyomonas gingivalis, P. endodontalis, Tannerella forsythia, Fusobacterium nucleatum, Pyramidobacter piscolens*
This study*	Culture, morphology, biochemistry, 16S rDNA cloning method	*Enterococcus faecalis, Streptococcus mutans, S. oralis, Lactobacillus fermentum, Rummeliibacillus stabekisii, * ***Clostridium hastiforme*** *, Parvimonas micra, Dialister invisus, Actinomyces oris, Propionibacterium acnes, * ***Corynebacterium minutissimum*** *, Rothia dentocariosa, * ***Neisseria elongata*** *, * ***Proteus hauseri***	***Enterococcus gallinarum*** */casseliflavus, * ***Lactobacillus gasseri*** *, Streptococcus sp., S. mutans, Selenomonas sp., Peptostreptococcus stomatis, Olsenella profusa, * ***Proteus hauseri/vulgaris*** *, Delftia sp., * ***Exiguobacterium aurantiacum, Pantoea agglomerans,*** * Neisseria sp* ***., Phocaeicola abscessus,*** * Candida parapsilosis*

* Taxa are listed as follows: Firmicutes, Actinobacteria, Spirochaetes, Proteobacteria, Bacteroidetes, Fusobacteria, Synergistes, Fungi.

** Bold: New taxa not found in any other study cited in Table 3.

Of the 7 phyla identified in root-filled teeth to date, 4, i.e. Firmicutes, Actinobacteria, Proteobacteria and Bacteroidetes are represented in this study and their frequency mirrors the one reported in the literature. In the present study, 13 of the detected bacterial taxa were isolated from or detected in treated root canals for the first time. Representatives of the genera *Enterococcus, Lactobacillus*, *Olsenella*, *Actinomyces*, *Neisseria, Clostridium* and *Corynebacterium* have been detected in treated root canals before, but not the same species as detected in the present study or not to the species level [8], [12], [16]. Different members of *Enterobacteriaceae, Bacteroidales* and *Bacillales* have been found frequently as well [5], [6], [12]. Yet *Delftia* sp., *Pantoea agglomerans, Proteus hauseri/vulgaris, Phocaeicola abscessus*, *Exiguobacterium aurantiacum* and *Rummeliibacillus stabekisii* have not been reported in previous studies. Most of these species have been described as either opportunistic pathogens or true pathogens in different human infections. *Delftia acidovorans* (also *Comamonas acidovorans*) and *Delftia tsuruhatensis* have been isolated in ocular infections, endocarditis and catheter-related infections [42], [43]. *Phocaeicola abscessus* is an obligate anaerobe species, belonging to the phylum Bacteroidetes that has been previously detected in a brain abscess [44]. *Exiguobacterium aurantiacum* was detected with denaturing gradient gel electrophoresis in periodontitis patients, and has also been isolated from blood cultures of patients with bacteremia [45], [46]. *Rummeliibacillus stabekisii*, bacilli related to *Bacillus pycnus* and the genus *Kurthia*, has only been described in 2009 as novel genus and novel species. and has not been reported in clinical infections yet [47].

These findings show that the diversity not only of primary endodontic infections but also of secondary infections is still greater than known to date. A previous molecular analysis using the 16S-rDNA cloning technique [16] showed a high species diversity and noted that often certain taxa were only found in a single case. The present study concurs with these results and points to an individual microbial profile for almost each treated root canal. This also indicates that the etiology of chronic apical periodontitis is far more heterogeneous than presumed by studies that used only cultural methods [48]. A previous study [17] that examined both root canal ends and periradicular tissue with culture-independent methods revealed bacteria in the majority of the periradicular tissue samples and showed diverse microbial profiles for the tissue and the root canal samples. The authors conclude that bacteria from a persistent biofilm on the root canal ends could invade into the surrounding periradicular tissue leading to a polymicrobial infection and to persistent periradicular lesions. It is particularly noteworthy that over half of the identified bacteria belonged to as-yet-uncultivated organisms. These findings are consistent with the results of the present study, suggesting that uncultivated phylotypes may contribute to persistent periradicular infections as a part of the microbial profile and might have been disregarded using cultural approaches.

It has been discussed earlier for caries and periodontitis that the microbial community profile present in the oral biofilms plays a bigger role in causing disease than actual single species [49]. This might also be true for endodontic infections [48]. Considering secondary endodontic infections as biofilm-associated disease it becomes of interest to examine how different species could synergize with each other. The present study revealed several cases with a multispecies infection. For example in one sample *Enterococcus faecalis*, *Exiguobacterium aurantiacum* and *Pantoea agglomerans* were detected. *E. faecalis* is known to be very resistant to the effects of chemomechanical preparation and at the same time capable of enduring low nutrient concentrations. In this way it could prepare the ground for the other two species. Another tooth harboured 12 different species, among them *Streptococcus mutans*, *S. oralis*, *S. salivarius*, *Lactobacillus fermentum*, *Actinomyces oris*, *Neisseria elongata*, *Selenomonas* sp., and *Peptostreptococcus stomatis*. A possible role of the *Neisseria* species could be to reduce the O_2_ concentration so that obligate anaerobes like *Selenomonas* sp. and *Peptostreptococcus stomatis* could establish. At the same time fermentation byproducts like CO_2_ produced by heterofermentative *Lactobacillus fermentum* could favor growth of *Actinomyces* species dependant on high CO_2_ concentrations. All these interactions are very intricate due to the complex metabolic pathways of the microorganisms involved and should be the subject of further studies. Even though in the present study culture and culture-independent methods revealed the same number of taxa, a greater diversity was found with the latter approach. Only the 16S-rDNA cloning technique detected one species belonging to the phylum Bacteroidetes and one fungal species. Fungi, especially *Candida* species, have often been found in molecular as well as cultural studies in up to 18% of root canal treated teeth [6]. Occasionally other yeast species, e.g. *Geotrichum* spp., *Rhodotorula* spp., and *Saccharomyces* spp. have been detected in primary infections [13].

In this study, 4 of the clones found with the 16S-rDNA cloning technique could only be identified to the genus level and represented as-yet-uncultivated phylotypes (*Selenomonas* sp., *Streptococcus* sp., *Delftia* sp. and *Neisseria* sp.). This result strengthens the assumption that up to 60% of the oral microorganisms cannot be cultivated [14] but may still play a significant role in the etiology of post-treatment apical periodontitis. The clone that belonged to the genus *Selenomonas* sp. showed a high percentage identity with a clone sequence that had been found in subgingival plaque [14]. The clone sequence that was identified as *Streptococcus* sp. matched a sequence that had been previously identified in an oropharyngeal sample [50] and the *Neisseria* sp. sequence showed very high similarity to one that had been found in sputum samples from cystic fibrosis patients [51]. Figure 1 shows the phylogenetic analysis of all taxa found with the 16S-rDNA cloning technique.

*Enterococcus faecalis*, which by some authors is accounted the most prevalent species associated with endodontic treatment failures, was found in no more than 2 cases and only with culture methods. This finding is in agreement with some studies [8], [16], [4] but in contrast to several others which detected *E. faecalis* in 30% to 89% of the positive samples [37], [52]. In previous studies, the specificity of root canal infections was discussed controversially. Several studies have found e.g. *Enterococcus faecalis* as the most prevalent species in filled root canals [38], [62] whereas others report a variety of species but no predominant one [4], [8], [16]. To date there is no consistent evidence for one conclusion or the other. Recent studies suggest that it is rather the bacterial community profile than certain specific species that are associated with different types of endodontic infections [48]. Our results revealed even more species that had not yet been found in filled root canals and did not suggest that there is a certain specific organism associated with secondary root canal infections. The composition of the microbial flora can also vary due to geographical locations or inter-individual and even nutritional differences [53]. In addition, variable canal treatments and irrigation procedures, as well as differences in coronal leakage and quality of temporary seals can play a role as well [1], [16]. These findings might argue that there is a slight overestimation of this species as suggested by Rôças et al. [54].

In summary, both culture methods and 16S-rDNA cloning technique revealed a high diversity of the microbiota including several new putative pathogenic microorganisms that had not been detected in root-filled teeth before (s. Table 3). The results of the culture-dependent and -independent methods for the most part did not overlap. Inherent differences in the methodology might have been the reason for this result, e.g. the open-ended PCR cloning method was able to reveal several as-yet-uncultivated microorganisms that escape cultural detection. On the other hand, the cloning method may have failed to detect some species due to loss of DNA and differential amplification.

Therefore, the authors favor the integration of data gained with both methods to complement each other and give a more comprehensive picture of the actual diversity of the endodontic flora. The findings suggest that the polymicrobial etiology of apical periodontitis is even more complex than assumed and that distinct bacterial communities possibly including as-yet-uncultivated taxa might be significant for it [48]. This fact should be taken into account when treatment protocols are devised. Up to now, sodium hypochloride at concentrations ranging from 0.5%–6%, ethylendiaminetetraacetic acid (EDTA), chlorhexidine (CHX) and Calcium hydroxide have been considered gold standards for the conventional chemomechanical preparation of the root canals [55], [56], [57]. Other alternative measures to eradicate resistant oral bacteria having survived root canal disinfection and intracanal medication include the use of apical negative pressure irrigation systems, ozone gas and photodynamic therapy [58], [59], [60], [61]. Passive ultrasonic irrigation also enables the elimination of oral biofilms adhering to root canal walls as well as bacteria located in isthmi and ramifications by mainly enhancing the bactericidal effects of root canal disinfectants [59]. Despite the plethora of chemomechanical preparation protocols the development of new root canal disinfection methods should serve the ultimate goal in endodontics: the sterilization of the root canal system.
